# Genomic Insights into Phosphorus Solubilization of *Pseudomonas extremaustralis*

**DOI:** 10.3390/microorganisms13040911

**Published:** 2025-04-16

**Authors:** Carolyn Mayer, Catherine Urrutia, Carol Jerez-Quezada, Patricio Javier Barra, Michel Abanto

**Affiliations:** 1 Facultad de Ciencias Agropecuarias y Medioambiente, Universidad de La Frontera, Temuco 4811230, Chile; c.mayer01@ufromail.cl (C.M.); c.urrutia08@ufromail.cl (C.U.); 2 Programa de Doctorado en Ciencias Mención Biología Celular y Molecular Aplicada, Universidad de La Frontera, Temuco 4811230, Chile; 3 Programa de Doctorado en Ciencias de Recursos Naturales, Universidad de La Frontera, Temuco 4811230, Chile; c.jerez02@ufromail.cl; 4 Center of Plant, Soil Interaction and Natural Resources Biotechnology, Scientific and Technological Bioresource Nucleus, Universidad de La Frontera, Temuco 4811230, Chile; patricio.barra@ufrontera.cl; 5 Genomics and Bioinformatics Unit, Scientific and Technological Bioresource Nucleus (BIOREN), Universidad de La Frontera, Temuco 4811230, Chile; 6 Biocontrol Research Laboratory, Universidad de La Frontera, Temuco 4811230, Chile

**Keywords:** *Pseudomonas extremaustralis*, phosphate-solubilizing bacteria, whole-genome sequencing, phosphorus-cycling genes

## Abstract

*Pseudomonas extremaustralis* was first isolated from Antarctica and gained interest for its ability to thrive in extreme environmental conditions and degrade recalcitrant compounds. Some strains have been identified as phosphobacteria, which play a significant role in phosphorus (P) cycling by solubilizing or mineralizing insoluble phosphate forms for plant uptake. However, there is limited knowledge about the genomic mechanisms involved in P-cycling in the species *P. extremaustralis*. In this study, we aimed to evaluate the genomic potential of *P. extremautralis* as a phosphobacteria species by screening genes related to P-cycling. Two *P. extremaustralis* strains from pisciculture sludge residues were selected to sequence their complete genomes based on their ability to solubilize inorganic P in vitro, and an in silico analysis with all the *P. extremaustralis* genomes was performed to identify the presence of phosphorus-cycling-related genes. Genes mainly involved in the metabolic processes of two-component systems and transporters, and genes involved in organic acid production and alkaline phosphatases, were identified. This study helps us to understand the metabolic potential of this species and its role as a solubilizer of phosphates and thus a facilitator of plant-available phosphorus, which could guide the use of this species of phosphobacteria in the development of sustainable agriculture.

## 1. Introduction

The genus *Pseudomonas* is well known for its metabolic versatility and ability to thrive in diverse and extreme environments, making it an important genus for biotechnological applications and environmental studies [1]. *Pseudomonas extremaustralis*, in particular, is a bacterium originally isolated for the first time from Antarctic environments, recognized for its robust resistance to environmental stressors and potential in bioremediation processes [2,3,4]. This species has garnered interest due to its ability to metabolize a wide range of substrates, including recalcitrant organic compounds, and its role in nutrient cycling [5].

The complete *P. extremaustralis* genomes available in the NCBI RefSeq database range in size between 6.1 and 7.2 Mb, with a G+C content of 60–61%. Genomic analyses have been focused on studying the mechanisms of these bacteria to endure environmental stressors, such as genes involved in the synthesis of polyhydroxyalkanoates (PHAs) [1], cold-shock proteins, and others [4].

Phosphorus is a critical nutrient in both aquatic and terrestrial ecosystems, and its availability often limits primary productivity [6]. The cycling of phosphorus in the environment involves various microbial processes, including the solubilization of inorganic phosphorus, which is essential for making this nutrient accessible to plants and other organisms [7]. Bacteria that can solubilize inorganic phosphorus play a vital role in enhancing soil fertility and promoting sustainable agriculture, particularly in phosphorus-deficient soils [8]. These bacteria are known as phosphobacteria; they mobilize inorganic phosphorus and, in other cases, organic phosphorus, for plant absorption through mechanisms such as the production of organic acids, phosphatases, and other compounds [7,9,10]. Using these bacteria in agricultural inoculants provides multiple advantages beyond enhancing phosphorus availability for plant uptake. Firstly, compared to traditional fertilization methods, these do not compromise soil health, making it a sustainable, long-term alternative [11]. Furthermore, their use is cost-effective, and their efficiency has been proven to enhance crop yield [8], thereby reducing dependence on chemical phosphate fertilizers, which are harmful to the environment when used in excess [12]. In this sense, the genomic characterization of efficient strains is a fundamental step in the design of optimized microbial consortia that will improve plant nutrition and soil health.

In this study, we isolated two strains of *P. extremaustralis* from pisciculture sludge, a nutrient-rich environment that likely selects microorganisms with enhanced nutrient cycling capabilities. Given the unique environmental conditions of their origin, we hypothesized that these strains may possess genes related to phosphorus cycling, which could be leveraged for biotechnological applications in nutrient management and sustainable agriculture. These strains were evaluated for their phosphate-solubilizing ability under in vitro conditions. We then conducted a comprehensive genomic analysis by sequencing their complete genomes and comparing them with other available *P. extremaustralis* genomes to identify genes involved in phosphorus cycling.

Given the limited knowledge about *P. extremaustralis* as a putative plant growth-promoting bacterium (PGPB) and its underexplored potential as a PSB, this study aims to fill the knowledge gap by uncovering novel mechanisms and pathways for phosphorus solubilization. Additionally, the unique origin of these strains from pisciculture sludge suggests that they may possess specific adaptations that confer superior phosphate-solubilizing abilities, making them promising candidates for agricultural applications. By advancing our understanding of the genomic basis of phosphorus solubilization in *P. extremaustralis*, this research could lead to innovative strategies for improving phosphorus use efficiency in agriculture, reducing reliance on chemical fertilizers, and mitigating their environmental impacts.

## 2. Materials and Methods

### 2.1. Isolation of Bacteria from Pisciculture Samples

Sludge and silage samples were obtained from pisciculture from the La Araucania region, Chile (39°15′10.4″ S 71°52′15.2″ W). Samples were inoculated into Luria–Bertani (LB) liquid medium and incubated at 32 °C for 2 days, shaking at 200 rpm. Samples were then isolated on MacConkey (MK) medium to select only Gram-negative bacteria and on mannitol salt medium to obtain only Gram-positive bacteria. Subsequently, to isolate pure strains, individual colonies were picked and isolated on tryptic soy agar (TSA) solid medium and grown overnight at 27 °C. The pure isolates were preserved in glycerol with LB liquid medium at −80 °C. To reactivate these isolates, they were transferred to 10 mL of liquid LB medium and incubated for 24 h at 26 °C with agitation at 120 rpm.

### 2.2. In Vitro Identification of Putative Phosphate-Solubilizing Bacteria

To screen for putative phosphate-solubilizing bacteria, the isolates were inoculated in triplicate onto the National Botanical Research Institute’s phosphate growth medium (NBRIP), including a control treatment. This medium contains tricalcium phosphate (Ca_3_(PO_4_)_2_), an inorganic form of phosphorus [13]. The plates were incubated at 27 °C for seven days; the results of this assay are positive if clear halos appear around the colonies.

### 2.3. Total DNA Extraction and Quantification

The isolates that tested positive in the NBRIP medium assay were further selected for whole-genome sequencing. Prior to DNA extraction, the strains were grown overnight in LB liquid medium at 27 °C with agitation. Then, total genomic DNA extraction was performed using the E.Z.N.A.^®^ Universal Pathogen Kit from OMEGA BIO-TEK (Norcross, GA, USA), following the manufacturer’s instructions. The concentration of the extracted DNA was measured using the Invitrogen Qubit 4 Fluorometer (Thermo Fisher Scientific, Inc., Waltham, MA, USA); to evaluate the integrity of the extracted DNA, electrophoresis on a 1% agarose gel was performed.

### 2.4. De Novo Sequencing and Genome Assembly

The extracted DNA was sent to Omega Bioservices (Norcross, GA, USA) for DNA library preparation and sequencing. The KAPA HyperPlus Kit (Roche, Basel, Switzerland) was used for library construction, and de novo whole sequencing was carried out using the Illumina MiSeq platform with 100× coverage. The quality of the raw reads was evaluated using FastQC (v.0.12.0) (http://www.bioinformatics.babraham.ac.uk/projects/fastqc/ (accessed on 11 November 2023)). Adapters and low-quality reads were trimmed using Fastp (v.0.23.4) [14] and Trimmomatic (v.0.39) [15]. De novo assembly was performed using Unicycler (v.0.5.0) [16], contigs shorter than 200 bp were removed, and genome annotation was carried out using Bakta (v.1.9.1) [17]. Both draft genome sequences were deposited in the NCBI database under BioProject accession number PRJNA1124157. The accession numbers are JBEOLZ000000000 for PeCHP2 and JBEOMA000000000 for PeCHP3.

### 2.5. Taxonomic Analysis

Once the genomes were obtained, they were uploaded to the Type (Strain) Genome Server (TYGS), available at https://tygs.dsmz.de/ (accessed on 11 June 2024). TYGS is a platform for conducting accurate genome-based taxonomy of prokaryotic genomes. It compares the genome uploaded with its database using the MASH algorithm to determine the closest type strain genome. It generates a phylogenetic analysis and performs digital DNA–DNA hybridization (dDDH) to estimate the relatedness of the genomes, allowing for accurate species identification [18]. The phylogenetic tree generated was visualized using iTOL [19].

### 2.6. Phylogenetic Analysis and Identification of Phosphorus-Cycling-Related Genes

A phylogenetic analysis using the TYGS platform [18] was performed using all *P. extremaustralis* genomes retrieved from the NCBI RefSeq database (https://www.ncbi.nlm.nih.gov/refseq/ (accessed on 17 August 2024)) to determine their evolutionary relationships, using the genome of *Pseudomonas veronii* DSM 11331 (GCF_001439695.1) as an outgroup. To identify genes potentially involved in the phosphorus cycle, we used the PCycDBv1.1 database, specialized for searching for genes related to the phosphorus cycle [20]; the database was used with the DIAMOND alignment tool [21]. Subsequently, we performed a sequence similarity search using DIAMOND BLASTx to compare all our genomes with the database. To filter the results and generate a list of potential candidate genes involved in the phosphorus cycle, we followed the recommendations of the PCyCDB database and applied a threshold of 30% identity, 30% alignment coverage, and a minimum hit length of 30 amino acids. Finally, a table was generated for each genome, describing each gene involved in the phosphorus cycle associated with each open reading frame (ORF) [20]. This search was also performed with the two plasmids found in each genome sequenced in this study.

Each table generated according to each genome was concatenated into a single file to be analyzed in R v.4.4.1 (https://www.r-project.org/ (accessed on 27 August 2024)) and converted to data frames to account for the frequency of each gene in each metabolic process, so a table was created containing each gene and the frequency in each genome, which was converted to a binary matrix of absence and presence that was combined with the phylogenetic tree of genomes in the iTOL platform v6.9.1 [19] in heatmap format. The metabolic processes of each gene were then added to the figure generated with the program Inkscape (https://inkscape.org/ (accessed on 16 September 2024)).

## 3. Results and Discussion

### 3.1. In Vitro Screening of Phosphate-Solubilizing Bacteria

The isolates were evaluated for their ability to solubilize inorganic phosphorus in the NBRIP medium. The results showed that, after seven days, the strains PeCHp-2 and PeCHp-3 developed clear zones around them in the NBRIP medium, meaning that they are possibly capable of solubilizing inorganic phosphate, specifically Ca_3_(PO_4_), by producing organic acids. These strains were selected to perform further genomic analysis.

### 3.2. Genetic Characterization Based on Whole-Genome Sequencing

By sequencing the strains PeCHp-2 and PeCHp-3, a total of 21.235.097 and 18.926.708 total raw reads, with a length of 151 bp, were obtained. After trimming low-quality reads and assembling them into contigs, a phylogenetic analysis based on the complete genome (Figure S1) was performed, confirming that the strains PeCHp-2 and PeCHp-3 belong to the species *Pseudomonas extremaustralis*, being the closest to *P. extremaustralis* 14-3 (91.8% sequence similarity), and that their G+C content corresponds to 60.7 mol%, respectively. The general features of the genomes, as obtained through Bakta annotation, are summarized in Table 1. Both genomes exhibit similar characteristics. When compared with other *P. extremaustralis* genomes available in the NCBI RefSeq database, their reported sizes range between 6.1 and 7.2 Mb, with a G+C content of approximately 60–61%. Each genome contains a single chromosome along with a plasmid of around 63.8 kbp, characterized by a G+C content of ~53.4 mol%. Notably, no pseudogenes, gaps, oriV, or oriT sequences were identified in either genome.

### 3.3. Phosphorus-Cycling Genes with P. extremaustralis Phylogenetic Analysis

A phylogenetic analysis was performed using the whole-genome sequences of all *P. extremaustralis* genomes available in the NCBI RefSeq database to determine their evolutionary relationships. This analysis used *Pseudomonas veronii* DSM 11331 (GCF_001439695.1) as the outgroup. The metadata associated with these genomes have been compiled and are provided in Table S1.

**Figure 1:**
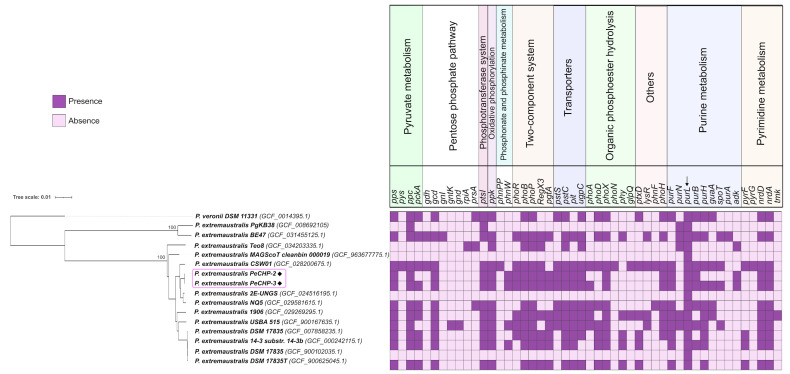
In silico screening of genes related to P-cycling with a phylogenetic tree based on whole genomes of *Pseudomonas extremaustralis* strains obtained from the NCBI RefSeq database. *P. veronii* was used as an outgroup, generated from the TYGS server. The assembly accession numbers are shown in parentheses. The scale bar represents a sequence divergence of 0.01; the values at the nodes represent the bootstrap support values. The strains marked in the pink square correspond to the strains PeCHP-2 and PeCHP-3, isolated and sequenced in this study. The arrow indicates the presence of this gene in all strains.

As shown in the phylogenetic tree in Figure 1, we can distinguish two main clades. The first clade includes *P. extremaustralis* PgKB38 (GCF_008692105.1) isolated from Panax ginseng from South Korea and *P. extremaustralis* BE47 (GCF_031455125.1) from extraradical hyphae from the United States. The d4 dDDH values of these genomes are below 38%; in comparison, for the other *P. extremaustralis,* the lowest d4 dDDH value corresponds to 83%, which means that the genomes *P. extremaustralis* PgKB38 (GCF_008692105.1) and *P. extremaustralis* BE47 (GCF_031455125.1) are significantly different from the other strains; dDDH values below 70% suggest that these genomes belong to different species [22]. In addition, these strains do not belong to any strains found in the TYGS server.

The strains PeCHp-2 and PeCHp-3 and the remaining genomes are on the second clade, with a bootstrap value of 100%. The closest genomes to the strains PeCHp-2 and PeCHp-3 are *P. extremaustralis* 2E-UNGS (GCF_024516195.1) isolated from Reconquista River, Argentina, which has been studied for its ability to form biofilms and self-aggregate for biotechnological applications [23], and *P. extremaustralis* CSW01 (GCF_028200675.1), which was isolated from activated sludge from Spain and has been described as having the ability to degrade high concentrations of paracetamol [24].

Among another clade formed by the genomes DSM 17835 (GCF_007858235.1), 14-3 (GCF_000242115.1), DSM 17835 (GCF_900102035.1), and DSM 17835T (GCF_900625045.1), the strain *P. extremaustralis* 14-3 was the first strain isolated from this species, isolated in 1994 from a temporary pond in Antarctica [2], and the strain *P. extremaustralis* DSM17835 (GCF_900102035.1), deposited at the German Collection of Microorganisms and Cell Cultures GmbH (Leibniz Institute DSMZ), corresponds to the same strain as *P. extremaustralis* 14-3; the closeness of the genomes and these findings suggest that these strains belonging in this clade are clonal.

As shown in the presence–absence matrix of P-cycling-related genes, all metabolic processes described in the PCycDB database are represented across the genomes analyzed. Notably, the *purL* gene (K23269), which encodes phosphoribosylformylglycinamidine synthase—an enzyme involved in purine metabolism—is present in all genomes, suggesting it may be a constitutive gene. Among the strains, *P. extremaustralis* CSW01 (GCF_028200675.1) and 1906 (GCF_029269295.1) exhibited the highest number of P-cycling genes, with 36 and 30 genes, respectively. These strains were isolated from distinct environments, sewage sludge in Spain, and oil sands tailings ponds in Canada. Interestingly, no publications were found that evaluate these strains as P-solubilizing bacteria, highlighting a potential avenue for future research.

The strains that contain the least number of these genes are *P. extremaustralis* DSM17835 (GCF_900102035.1), MAGScoT_cleanbin_000019 (GCF_963677775.1), and 2E-UNGS (GCF_024516195.1), containing only the *purl* (K23269) gene. By removing these genomes, including PgKB38 (GCF_008692105.1) and Teo8 (GCF_034203335.1), the genes that are common in the rest of the genomes are mentioned in Table 2, extracted from Table 1 of Additional File 1 of PCycDB: a comprehensive and accurate database for fast analysis of phosphorus cycling genes [20].

Among these, the gene *gcd* (K00117), which codes for quinoprotein glucose dehydrogenase (GDH), has been described as a reliable marker for identifying phosphate-solubilizing bacteria [25]; this enzyme is fundamental for inorganic phosphorus solubilization, by oxidizing glucose into gluconic acid, which lowers the pH of the soil [26], or by chelating ions that are bound to phosphorus [11]. Through the annotation of the PeCHP-2 and PeCHP-3 genomes, we found the *pqqABCDF* cluster, which contains genes responsible for the biosynthesis of pyrroloquinoline quinone (PQQ), an important cofactor for the enzyme GDH [27]. This may suggest that the strains PeCHP-2 and PeCHP-3 were able to solubilize inorganic phosphate in vitro by producing gluconic acid.

Regarding the isolates that were sequenced in this study, in the genome of *P. extremaustralis* PeCHP-2, the presence of 24 genes related to P-cycling was found, and while the strain PeCHP-3 has 28 genes, both strains have the majority of the genes involved in the two-component system (*phoR*, *phoB*, *phoP*, *RegX3*, and *pgtA*), which, under P depletion, regulates the expression of transporter genes and phosphatase genes [20] and is involved with transporters (*pstS*, *pstC*, *pit*, and *ugpC*).

Concerning the genes that encode for phosphatases, the gene *phoA* (K01077) (intracellular APase) is present in PeCHP-3, and the gene *phoX* (K07093) (extracellular APase) is present in both genomes; these genes code for alkaline phosphatases, enzymes that are responsible for the mineralization of organic P, found mainly in neutral or alkaline soils [8]. Regarding *phoX* proteins, they are distinct from the well-characterized *phoA* family and are more widely distributed in marine bacteria than classical PhoA proteins, observed in the responses of APase genes under environmental P conditions in *Microcystis aeruginosa*; the results showed that, in an organophosphate medium, the expression of *phoX* was higher than that of *phoA*, implying that *Microcystis* increased the extracellular secretion of APase to break down organic substrates in the medium. In a P-deficient medium, *phoA* could be induced to break down the poly-P substrates stored in the cells [28]. No P-cycling-related genes were found in PeCHP-2 and PeCHP-3 plasmids, suggesting that these genes are present in the chromosome.

The genera *Pseudomonas* has been described as the second most predominant genera among P-solubilizing bacteria [12]; regarding the species *P. extremaustralis*, previous studies describe some *P. extremaustralis* strains as phosphate solubilizers [29,30,31], along with having other PGPB traits like auxin production [31], nitrogen fixation [32], and indole-3-acetic acid (IAA) production [30]. In this study, the two *P. extremaustralis* strains solubilize inorganic phosphate in vitro, and genes involved in P-cycling were found in their genomes. These results are consistent with earlier studies. However, it is necessary to evaluate these strains using in situ analysis to confirm these in vitro and in silico results.

This species is particularly interesting as a PGPB due to its ability to thrive in extreme environmental conditions, such as low temperatures [33], and the capacity to degrade recalcitrant hydrocarbons, which is associated with its ability to produce polyhydroxyalkanoates [34], allowing the formation of biofilms and oxidative stress response [35,36]. These properties may allow them to still function as phosphobacteria under unfavorable environmental conditions. To confirm if the strains PeCHP-2 and PeCHP-3 possess these properties, further studies need to be performed. Genomic analyses of the species *P. extremaustralis* have been conducted before with a focus on exploring their genetic mechanism allowing this species to adapt to stressful environmental conditions [4]. To our knowledge, this is the first genomic analysis of the species *P. extremaustralis* focused on phosphorus solubilization traits. These studies are relevant to understanding P-cycling in the soil and exploring microorganisms and molecules with industrial applications [37].

## 4. Conclusions

In this study, two strains, PeCHP-2 and PeCHP-3, isolated from pisciculture sludge were identified as belonging to the *Pseudomonas extremaustralis* species. These strains were selected due to their ability to solubilize inorganic phosphate in vitro to sequence their complete genome, and this allows us to perform a comprehensive genomic analysis of the *P. extremaustralis* species, focusing on P-cycling genes. Mainly genes involved in metabolic processes like the two-component system and purine metabolism, genes encoding alkaline phosphatase, and the production of organic acids were found. These genes are crucial for inorganic phosphorus solubilization and modulation of other metabolic processes under P depletion. Some *P. extremaustralis* strains have been previously described as phosphate-solubilizing bacteria based on in vitro and, in some cases, in vivo assays. However, in these studies, genomic analyses were only focused on 16S rRNA gene identification or genes involved in other properties. Here, we identified and evaluated the genetic determinants related to the phosphate solubilization potential of these bacteria, offering a deeper understanding of the mechanisms involved in phosphorus solubilization. Providing valuable insight into the genetic potential of this species could lead to the development of strategies toward more sustainable agriculture.

## Figures and Tables

**Table 1:** General characteristics of the genomes *P. extremaustralis* PeCHP-2 and PeCHP-3.

Strain	PeCHP-2	PeCHP-3
Name	*Pseudomonas extremaustralis*	*Pseudomonas extremaustralis*
Length (bp)	6,192,902	6,201,072
Contigs	176	178
G+C (%)	60.7	60.7
N50	115,271	109,599
L50	15	19
Plasmids	1 (63,855 bp)	1 (63,704 bp)
Annotation		
tRNA	60	60
tmRNA	2	2
rRNA	3	2
ncRNA	48	48
ncRNA regions	35	35
CRISPR	0	1
CDS	5601	5601
ORFs	5	5
Hypothetical	559	559
oriC	1	1

**Table 2:** Genes related to phosphorus cycling present in all *P. extremaustralis* genomes, except those with the lowest number of genes.

Metabolic Process	Genes	KO Number	Function
Pyruvate metabolism	*pps*	K01007	Pyruvate, water dikinase
	*ppc*	K01595	Phosphoenolpyruvate carboxylase
Pentose phosphate pathway	*gcd*	K00117	Quinoprotein glucose dehydrogenase
Phosphotransferase system	*ptsl*	K08483	Phosphoenolpyruvate–protein phosphotransferase
Oxidative phosphorylation	*ppk*	K00937	Polyphosphate kinase
Two-component system	*phoB*	K07657	Two-component system, OmpR family, phosphate regulon response regulator PhoB
	*phoP*	K07658, K07660	Two-component system, OmpR family, alkaline phosphatase synthesis response regulator PhoP
Transporters	*pstC*	K02037	Phosphate transport system permease protein
Organic phosphoester hydrolysis	*phoX*	K07093	Alkaline phosphatase
Purine metabolism	*purL*	K23269	Phosphoribosylformylglycinamidine synthase
Pyrimidine metabolism	*nrdD*	K21636	Ribonucleoside-triphosphate reductase
	*nrdA*	K00525	Ribonucleoside-diphosphate reductase alpha chain

## Data Availability

The genomic data and associated information used in this research were retrieved from the NCBI database (https://www.ncbi.nlm.nih.gov) and can be accessed through the accession numbers described in the Supplementary Table.

## Supplementary Materials

The following supporting information can be downloaded at https://www.mdpi.com/article/10.3390/microorganisms13040911/s1, Figure S1: Taxonomic identification based on complete genome information inferred by Type Strain Genome Server (TYGS); Table S1: Metadata of *Pseudomonas extremaustralis* genomes extracted from the NCBI RefSeq database.
