# Supplementary material for: Genomic Insights into Phosphorus Solubilization of Pseudomonas extremaustralis
Source: Microorganisms. 2025 Apr 16;13(4):911. doi: 10.3390/microorganisms13040911 (PMC12029462; doi:10.3390/microorganisms13040911)
Supplement: Supplementary file 1 [file microorganisms-13-00911-s001.zip › Table_S1.pdf]

Genomic insights into phosphorus solubilisation of *Pseudomonas extremaustralis*

Carolyn Mayer [1,2], Catherine Urrutia [1,2], Carol Jerez-Quezada [3,4,5], Patricio Javier Barra [4,6] and Michel Abanto [4,5,*]

[1] Facultad de Ciencias Agropecuarias y Medioambiente, Universidad de La Frontera, Temuco 4811230, Chile
[2] Programa de Doctorado en Ciencias Mención Biología Celular y Molecular Aplicada, Universidad de La Frontera, Temuco 4811230, Chile
[3] Programa de Doctorado en Ciencias de Recursos Naturales, Universidad de La Frontera, Temuco 4811230, Chile
[4] Center of Plant, Soil Interaction and Natural Resources Biotechnology, Scientific and Technological Bioresource Nucleus, Universidad de La Frontera, Temuco 4811230, Chile
[5] Genomics and Bioinformatics Unit, Scientific and Technological Bioresource Nucleus (BIOREN), Universidad de La Frontera, Temuco 4811230, Chile
[6] Biocontrol Research Laboratory, Universidad de La Frontera, Temuco 4811230, Chile
* Correspondence: mfabanto@gmail.com; Tel.: +56-452596520

Table S1. Metadata of *Pseudomonas extremaustralis* genomes recovered from the NCBI Refseq database

Assembly	GenBank	RefSeq	Strain	Submitter	Size (Mb)	Level	N50	L50	GC%	Genome coverage	Nº contigs /scaffolds	Host	Collection date	Geographic location	Sequencing technology
IMG-taxon 2667527225 annotated assembly	GCA_900102035.1	GCF_900102035.1	DSM 17835	DOE - JOINT GENOME INSTITUTE	6.7	Chromosome	6.7 Mb	1	60.5	67.0x	1	Not applicable	Not collected	Missing	PacBio RS
ASM2451619v1	GCA_024516195.1	GCF_024516195.1	2E-UNGS	Universidad Nacional General Sarmiento, Argentina	6.4	Chromosome	6.4 Mb	1	61	32.6x	1	River sediments	2004-03	Argentina: Rio Reconquista, Buenos Aires	Sanger
ASM785823v1	GCA_007858235.1	GCF_007858235.1	DSM 17835	Technische Universitaet Muenchen	6.6	Contig	187.8 kb	106	60.5	41.0x	11	Temporary water pond	1994-02	Antarctica	Illumina MiSeq
DSM_17835T	GCA_900625045.1	GCF_900625045.1	DSM 17835T	CEBITEC	6.6	Contig	182.3 kb	152	60.5	40.0x	11	Meat	2017	Germany	Missing
Pext14-3B_1.0	GCA_000242115.2	GCF_000242115.1	14-3 substr. 14-3b	INDEAR	6.6	Contig	135.7 kb	135	60.5	20.0x	13	Temporary water pond in Antarctica	1994	Argentina: Antarctica	454 GS-FLX Titanium

ASM3420333v1	GCA_034203335.1	GCF_034203335.1	Teo8	Georg-August-University Goettingen	7.1	Contig	5.9 Mb	14	60.5	350.0x	1	Activated sludge	2021-01-15	Germany	Illumina MiSeq;Oxford Nanopore MinION
ASM869210v1	GCA_008692105.1	GCF_008692105.1	PgKB38	Korea Research Institute of Bioscience and Biotechnology	7.1	Contig	4.1 Mb	8	60.5	93.0x	1	Panax ginseng	2017	South Korea	PacBio RSII; Illumina HiSeq
ASM3145512v1	GCA_031455125.1	GCF_031455125.1	BE47	DOE Joint Genome Institute	7.2	Scaffold	225.9 kb	68	60.5	207.0x	13	Extraradical hyphae	2016	USA: Ithaca, New York	Illumina
ASM2958161v1	GCA_029581615.1	GCF_029581615.1	NQ5	Texas A&M University	6.5	Contig	205 kb	95	60.5	33.0x	13	Membrane bioreactor	2022	USA: New Jersey	Illumina MiSeq
IMG-taxon 2671180025 annotated assembly	GCA_900167635.1	GCF_900167635.1	USBA 515	DOE - JOINT GENOME INSTITUTE	6.1	Scaffold	158.9 kb	69	61	239.0x	14	Superparamo soil	2010	Colombian Andes	Illumina HiSeq 2500
ASM2926929v1	GCA_029269295.1	GCF_029269295.1	1906	Universite Laval	6.3	Contig	114.6 kb	121	61	50.0x	15	Oil sands tailings pond	2013-06-01	Canada: Alberta	Illumina MiSeq
ASM2820067v1	GCA_028200675.1	GCF_028200675.1	CSW01	Instituto de Recursos Naturales y Agrobiologia de Sevilla. CSIC	7.1	Scaffold	84.1 kb	199	60	202.0x	24	Sewage sludge	2021-12	Spain: Seville	Illumina NovaSeq
piPlaSpea1.Pseudomonas_extremaustrali	GCA_963677775.1	GCF_963677775.1	6328a48c-d287-402b-9a22-4be284e47c5f	WELLCOME SANGER INSTITUTE	6.3	Complete	6.3 Mb	1	61	20.0x	1	Plagiopyla sp. strain OYSTR	2022-09-02	USA	Pacbio Sequel II
