# Supplementary figures and images for: Genomic Insights into Phosphorus Solubilization of Pseudomonas extremaustralis
Source: Microorganisms. 2025 Apr 16;13(4):911. doi: 10.3390/microorganisms13040911 (PMC12029462; doi:10.3390/microorganisms13040911)

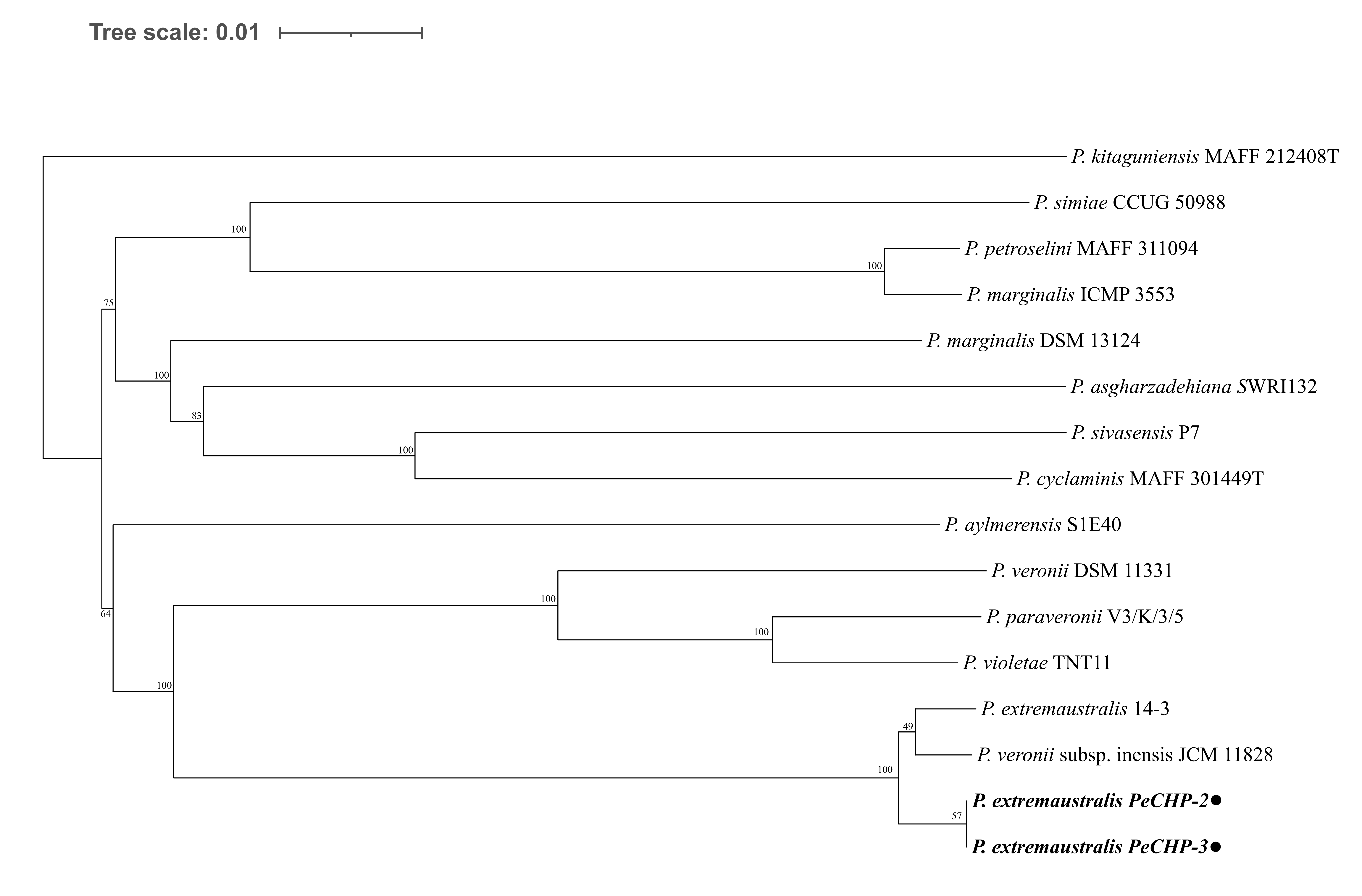

Supplement: Supplementary file 1 [file microorganisms-13-00911-s001.zip › Figure_S1.png]
